# *N*′-(2-Chloro­benzyl­idene)-2-(3,4-dimethyl-5,5-dioxo-2*H*,4*H*-pyrazolo­[4,3-*c*][1,2]benzothia­zin-2-yl)acetohydrazide

**DOI:** 10.1107/S160053681005227X

**Published:** 2010-12-24

**Authors:** Matloob Ahmad, Hamid Latif Siddiqui, Manzoor Iqbal Khattak, Saeed Ahmad, Masood Parvez

**Affiliations:** a Institute of Chemistry, University of the Punjab, Lahore 54590, Pakistan; c Department of Chemistry, University of Baluchistan, Quetta 6700, Pakistan; b Applied Chemistry Research Centre, PCSIR Laboratories Complex, Lahore 54600, Pakistan; d Department of Chemistry, Gomal University, Dera Ismail Khan, Pakistan; e Department of Chemistry, The University of Calgary, 2500 University Drive NW, Calgary, Alberta, Canada T2N 1N4

## Abstract

The asymmetric unit of the title compound, C_20_H_18_ClN_5_O_3_S, contains two independent mol­ecules with significantly different conformations of the heterocyclic thia­zine rings. In both mol­ecules, the thia­zine rings adopt half-chair conformations, with the S atoms displaced by 0.382 (3) and 0.533 (3) Å and N atoms −0.351 and −0.275 Å, respectively, from the planes formed by the remaining ring atoms. The crystal structure is stabilized by weak inter­molecular N—H⋯O and C—H⋯O inter­actions.

## Related literature

For related structures, see: Ahmad *et al.* (2008 ▶, 2009 ▶, 2011 ▶); Siddiqui *et al.* (2008 ▶). For puckering parameters, see: Cremer & Pople (1975 ▶).
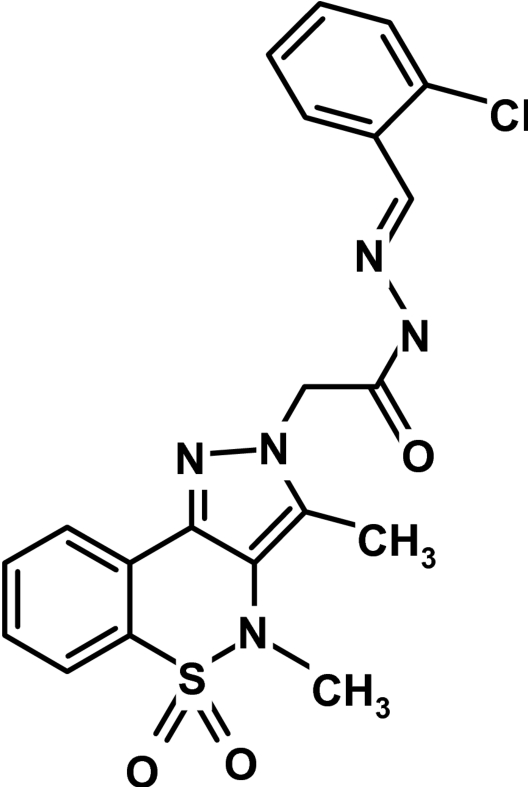


## Experimental

### 

#### Crystal data

C_20_H_18_ClN_5_O_3_S
*M* *_r_* = 443.90
Triclinic, 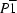

*a* = 11.4881 (2) Å
*b* = 12.7518 (3) Å
*c* = 15.5690 (4) Å
α = 71.2778 (11)°
β = 78.6837 (13)°
γ = 70.4911 (12)°
*V* = 2025.92 (8) Å^3^
*Z* = 4
Mo *K*α radiation
μ = 0.33 mm^−1^
*T* = 173 K
0.20 × 0.18 × 0.16 mm

#### Data collection

Nonius KappaCCD diffractometer
Absorption correction: multi-scan (*SORTAV*; Blessing, 1997 ▶) *T*_min_ = 0.938, *T*_max_ = 0.950
13799 measured reflections
7106 independent reflections
5649 reflections with *I* > 2σ(*I*)
*R*_int_ = 0.024

#### Refinement

*R*[*F* ^2^ > 2σ(*F* ^2^)] = 0.036
*wR*(*F* ^2^) = 0.106
*S* = 1.00
7106 reflections
545 parameters
H-atom parameters constrained
Δρ_max_ = 0.25 e Å^−3^
Δρ_min_ = −0.39 e Å^−3^

### 

Data collection: *COLLECT* (Hooft, 1998 ▶); cell refinement: *DENZO* (Otwinowski & Minor, 1997 ▶); data reduction: *SCALEPACK* (Otwinowski & Minor, 1997 ▶); program(s) used to solve structure: *SHELXS97* (Sheldrick, 2008 ▶); program(s) used to refine structure: *SHELXL97* (Sheldrick, 2008 ▶); molecular graphics: *ORTEP-3* for Windows (Farrugia, 1997 ▶); software used to prepare material for publication: *SHELXL97*.

## Supplementary Material

Crystal structure: contains datablocks global, I. DOI: 10.1107/S160053681005227X/jh2243sup1.cif

Structure factors: contains datablocks I. DOI: 10.1107/S160053681005227X/jh2243Isup2.hkl

Additional supplementary materials:  crystallographic information; 3D view; checkCIF report

## Figures and Tables

**Table 1:** Hydrogen-bond geometry (Å, °)

*D*—H⋯*A*	*D*—H	H⋯*A*	*D*⋯*A*	*D*—H⋯*A*
N4—H04⋯O3^i^	0.88	2.01	2.883 (2)	172
N9—H09⋯O6^ii^	0.88	1.96	2.837 (2)	173
C18—H18⋯O1^iii^	0.95	2.55	3.204 (3)	127
C29—H29*A*⋯O2^iv^	0.98	2.31	3.266 (3)	165
C38—H38⋯O4^v^	0.95	2.54	3.233 (2)	130

Symmetry codes: (i) 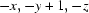; (ii) 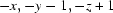; (iii) 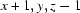; (iv) 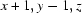; (v) 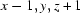.

**Fig. 1.**
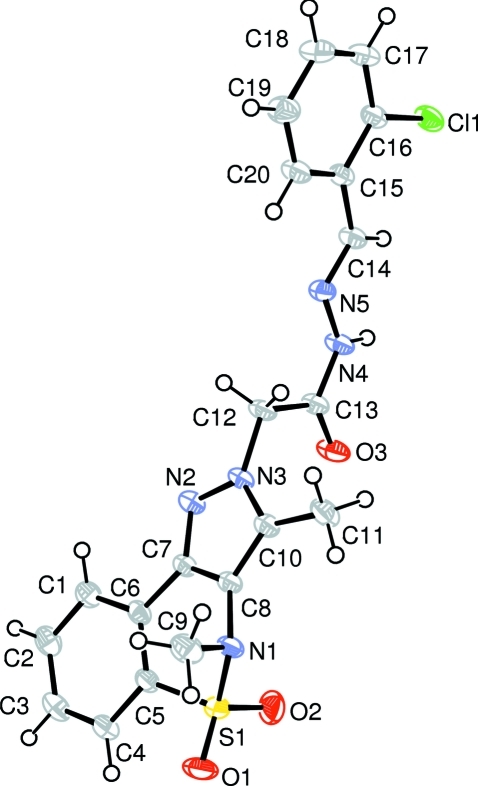
The molecule a plotted with the displacement ellipsoids at 50% probability level (Farrugia, 1997).

**Fig. 2.**
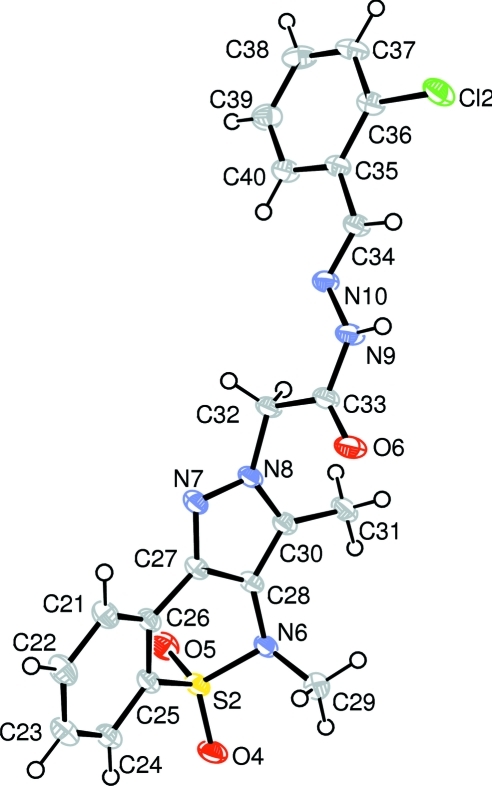
The molecule b plotted with the displacement ellipsoids at 50% probability level (Farrugia, 1997).

## supplementary crystallographic information

### Comment

In continuation to our research exploring potential biologically active derivatives of benzothiazines (Ahmad *et al.*, 2008; 2009), we have devised the fusion of the pyrazole moiety with 1,2-benzothiazine nucleus in an attempt to synthesize novel bioactive molecules. In this paper, we report the synthesis and crystal structure of the title compound, (I).

In the structure of the title compound, there are two independent molecules (molecule a and molecule b) in an asymmetric unit with significantly different conformations of the heterocyclic thiazine rings (Figs. 1 and 2). In both molecules, the thiazine rings adopt half-chair conformations. In molecule a, S1 and N1 atoms are displaced by 0.382 (3) and -0.351 (3) Å, respectively, from the plane formed by the remaining ring atoms(C5–C8). In molecule b, S2 and N6 atoms are displaced by 0.533 (3) and -0.275 (3) Å, respectively, from the plane formed by the remaining ring atoms(C25–C28). The methyl groups attached to N1 and N6 are displaced by significantly different distances from the basal planes of the thiazine rings in the two molecules; 1.802 (4) and 1.694 (4) Å, respectively. The pertinent puckering parameters (Cremer & Pople, 1975) in molecules a and b are: *Q* = 0.475 (2) and 0.532 (4) Å, θ = 60.0 (4) and 63.4 (2)° and φ = 27.8 (3) and 20.0 (2)°, respectively. Similar conformations of the corresponding rings have been reported in some closely related molecules (Siddiqui *et al.*, 2008; Ahmad *et al.*, 2011).

The mean-planes defined by the pyrazolo and benzene rings of the benzothiazin fragment are inclined with respt to each other at 12.55 (7) and 18.04 (8)°, in the molecules a and b, respectively. The chlorophenyl-methylidene-acetohydrazide moieties in the two molecules display identical conformation. In the molecules labeled as a, intermolecular hydrogen bonds N4—H04···O3 result in the formation of dimmers and C18—H18···O1 link the molecules into chains. Similarly, the molecules b also exhibit intermolecular hydrogen bonds N9—H09···O6 resulting in the formation of dimmers while C38—H38···O4 link the molecules into chains. The molecules a and b are connected *via* C29—H29B···O2 hydrogen bonds further stabilizing the crystal structure (Tab. 1).

### Experimental

A mixture of 2-(3,4-dimethyl-5,5-dioxidopyrazolo[4,3-*c*][1,2]benzothiazin- 2(4*H*)-yl)acetohydrazide (1.0 g, 3.12 mmol) and 2-chlorobenzaldehyde (0.44 g, 3.12 mmol) were dissolved in ethanol (50 ml) followed by the addition of 2 drops of glacial acetic acid. The mixture was subjected to reflux for 4 - 5 h. The completion of reaction was monitored with the help of thin layer chromatography (TLC). The precipitates formed were collected and washed with methanol (yield = 77%). The crystals of (I) suitable for crystallographic analysis were grown from its solution in dimethylamide at room temperature by slow evaporation.

### Refinement

All the H atoms were discernible in the difference electron density map. However, they were positioned at the idealized positions and refined by the riding-model approximation using constraints: N—H = 0.88 Å, C—H = 0.98, 0.99 and 0.95 Å for methyl, methylene and aryl H-atoms, respectively, and *U*_iso_(H) = 1.2*U*_eq_(parent atoms). The methyl groups were allowed to rotate about their axes during the refinement.

### Crystal data

**Table:**

C_20_H_18_ClN_5_O_3_S	*Z* = 4
*M**_r_* = 443.90	*F*(000) = 920
Triclinic, *P*1	*D*_x_ = 1.455 Mg m^−^^3^
Hall symbol: -P 1	Mo *K*α radiation, λ = 0.71073 Å
*a* = 11.4881 (2) Å	Cell parameters from 8973 reflections
*b* = 12.7518 (3) Å	θ = 1.0–27.5°
*c* = 15.5690 (4) Å	µ = 0.33 mm^−^^1^
α = 71.2778 (11)°	*T* = 173 K
β = 78.6837 (13)°	Block, colorless
γ = 70.4911 (12)°	0.20 × 0.18 × 0.16 mm
*V* = 2025.92 (8) Å^3^	

### Data collection

**Table:**

Nonius KappaCCD diffractometer	7106 independent reflections
Radiation source: fine-focus sealed tube	5649 reflections with *I* > 2σ(*I*)
graphite	*R*_int_ = 0.024
ω and φ scans	θ_max_ = 25.0°, θ_min_ = 1.4°
Absorption correction: multi-scan (*SORTAV*; Blessing, 1997)	*h* = −13→13
*T*_min_ = 0.938, *T*_max_ = 0.950	*k* = −15→15
13799 measured reflections	*l* = −18→18

### Refinement

**Table:**

Refinement on *F*^2^	Primary atom site location: structure-invariant direct methods
Least-squares matrix: full	Secondary atom site location: difference Fourier map
*R*[*F*^2^ > 2σ(*F*^2^)] = 0.036	Hydrogen site location: difference Fourier map
*wR*(*F*^2^) = 0.106	H-atom parameters constrained
*S* = 1.00	*w* = 1/[σ^2^(*F*_o_^2^) + (0.061*P*)^2^ + 0.760*P*] where *P* = (*F*_o_^2^ + 2*F*_c_^2^)/3
7106 reflections	(Δ/σ)_max_ = 0.001
545 parameters	Δρ_max_ = 0.25 e Å^−^^3^
0 restraints	Δρ_min_ = −0.39 e Å^−^^3^

### Special details

**Table:**

Experimental. Colorless crystals; mp 482 - 484 K. IR (KBr) cm^-1^: 3478, 1699, 1595, 1340, 1155. ^1^H-NMR (DMSO– d_6_) (400 MHz) δ 2.32 (3*H*, s, CC*H*_3_), 2.98 (3*H*, s, NC*H*_3_), 5.52 (2*H*, s, NC*H*_2_), 7.63–7.69 (2*H*, m, Ar*H*), 7.76–7.80 (2*H*, q, J = 17.9, 7.6 Hz, Ar*H*), 7.86 (1*H*, d, J = 7.80 Hz, Ar*H*), 7.93 (1*H*, d, J = 7.7 Hz, Ar*H*), 8.08 (1*H*, d, J = 8.2 Hz, Ar*H*), 8.16 (1*H*, d, J = 8.0 Hz, Ar*H*), 8.46 (1*H*, s, N═C*H*), 12.09 (1*H*, br s, N*H*). ^13^C NMR: 8.5, 38.9, 51.3, 121.4, 122.4, 123.1, 124.6, 124.7, 124.9, 126.2, 126.4, 127.5, 127.7, 127.8, 128.1, 130.6, 132.9, 134.2, 136.2, 136.8, 166.3. MS m/*z*: 444.0(*M*^+^).
Geometry. All e.s.d.'s (except the e.s.d. in the dihedral angle between two l.s. planes) are estimated using the full covariance matrix. The cell e.s.d.'s are taken into account individually in the estimation of e.s.d.'s in distances, angles and torsion angles; correlations between e.s.d.'s in cell parameters are only used when they are defined by crystal symmetry. An approximate (isotropic) treatment of cell e.s.d.'s is used for estimating e.s.d.'s involving l.s. planes.
Refinement. Refinement of *F*^2^ against ALL reflections. The weighted *R*-factor *wR* and goodness of fit *S* are based on *F*^2^, conventional *R*-factors *R* are based on *F*, with *F* set to zero for negative *F*^2^. The threshold expression of *F*^2^ > σ(*F*^2^) is used only for calculating *R*-factors(gt) *etc*. and is not relevant to the choice of reflections for refinement. *R*-factors based on *F*^2^ are statistically about twice as large as those based on *F*, and *R*- factors based on ALL data will be even larger.

### Fractional atomic coordinates and isotropic or equivalent isotropic displacement parameters (Å^2^)

**Table:**

	*x*	*y*	*z*	*U*_iso_*/*U*_eq_	
Cl1	0.25790 (5)	0.47702 (5)	−0.33819 (4)	0.03347 (14)	
Cl2	−0.26345 (5)	−0.48073 (5)	0.84350 (4)	0.03564 (15)	
S1	−0.50025 (5)	0.25138 (5)	0.37138 (3)	0.03121 (15)	
S2	0.50831 (4)	−0.18110 (4)	0.15070 (3)	0.02402 (13)	
O1	−0.61045 (14)	0.22302 (16)	0.41864 (11)	0.0472 (4)	
O2	−0.48908 (16)	0.36345 (13)	0.35969 (11)	0.0462 (4)	
O3	−0.11061 (14)	0.42389 (13)	0.08697 (10)	0.0364 (4)	
O4	0.61488 (12)	−0.22162 (13)	0.09197 (10)	0.0330 (3)	
O5	0.50899 (13)	−0.10151 (12)	0.19769 (10)	0.0305 (3)	
O6	0.10093 (14)	−0.41879 (13)	0.41403 (10)	0.0336 (4)	
N1	−0.48174 (14)	0.23141 (14)	0.26951 (11)	0.0256 (4)	
N2	−0.14910 (15)	0.17558 (14)	0.21567 (11)	0.0257 (4)	
N3	−0.19344 (14)	0.23215 (14)	0.13286 (11)	0.0235 (4)	
N4	0.00120 (15)	0.38951 (15)	−0.04161 (11)	0.0271 (4)	
H04	0.0281	0.4500	−0.0526	0.033*	
N5	0.03678 (15)	0.32466 (14)	−0.10331 (11)	0.0247 (4)	
N6	0.47766 (14)	−0.29427 (14)	0.22751 (11)	0.0232 (4)	
N7	0.15457 (14)	−0.16124 (14)	0.29530 (11)	0.0240 (4)	
N8	0.19665 (14)	−0.23898 (14)	0.37419 (11)	0.0226 (4)	
N9	−0.00865 (15)	−0.38815 (15)	0.54391 (11)	0.0264 (4)	
H09	−0.0389	−0.4461	0.5523	0.032*	
N10	−0.04085 (14)	−0.32727 (14)	0.60840 (11)	0.0235 (4)	
C1	−0.15139 (19)	0.05007 (17)	0.41734 (14)	0.0271 (4)	
H1	−0.0729	0.0304	0.3834	0.033*	
C2	−0.1625 (2)	0.00523 (19)	0.51109 (14)	0.0317 (5)	
H2	−0.0914	−0.0450	0.5409	0.038*	
C3	−0.2761 (2)	0.03255 (19)	0.56234 (15)	0.0339 (5)	
H3	−0.2822	0.0019	0.6268	0.041*	
C4	−0.3804 (2)	0.10447 (18)	0.51930 (14)	0.0318 (5)	
H4	−0.4588	0.1226	0.5537	0.038*	
C5	−0.36949 (18)	0.14994 (17)	0.42513 (13)	0.0259 (4)	
C6	−0.25467 (17)	0.12395 (16)	0.37233 (13)	0.0234 (4)	
C7	−0.25122 (17)	0.17367 (16)	0.27358 (13)	0.0214 (4)	
C8	−0.35875 (17)	0.22887 (17)	0.22753 (13)	0.0226 (4)	
C9	−0.5213 (2)	0.1342 (2)	0.26466 (16)	0.0358 (5)	
H9A	−0.5154	0.1350	0.2008	0.043*	
H9B	−0.6074	0.1426	0.2917	0.043*	
H9C	−0.4674	0.0607	0.2983	0.043*	
C10	−0.31995 (17)	0.26745 (17)	0.13709 (13)	0.0237 (4)	
C11	−0.3911 (2)	0.3342 (2)	0.05664 (14)	0.0342 (5)	
H11A	−0.4802	0.3497	0.0762	0.041*	
H11B	−0.3686	0.2891	0.0122	0.041*	
H11C	−0.3713	0.4076	0.0286	0.041*	
C12	−0.10719 (18)	0.25138 (18)	0.05219 (13)	0.0264 (4)	
H12A	−0.1446	0.2551	−0.0012	0.032*	
H12B	−0.0307	0.1852	0.0593	0.032*	
C13	−0.07352 (18)	0.36260 (18)	0.03483 (13)	0.0256 (4)	
C14	0.10219 (17)	0.36567 (18)	−0.17459 (13)	0.0257 (4)	
H14	0.1229	0.4339	−0.1801	0.031*	
C15	0.14552 (17)	0.30888 (17)	−0.24767 (13)	0.0239 (4)	
C16	0.21805 (17)	0.35203 (18)	−0.32574 (14)	0.0262 (4)	
C17	0.25943 (19)	0.2978 (2)	−0.39504 (15)	0.0339 (5)	
H17	0.3080	0.3289	−0.4479	0.041*	
C18	0.2294 (2)	0.1983 (2)	−0.38658 (15)	0.0375 (5)	
H18	0.2584	0.1601	−0.4334	0.045*	
C19	0.1572 (2)	0.1542 (2)	−0.31005 (15)	0.0355 (5)	
H19	0.1366	0.0859	−0.3044	0.043*	
C20	0.11527 (19)	0.20949 (19)	−0.24212 (14)	0.0300 (5)	
H20	0.0646	0.1793	−0.1904	0.036*	
C21	0.15474 (18)	−0.05797 (17)	0.08887 (14)	0.0264 (4)	
H21	0.0741	−0.0537	0.1200	0.032*	
C22	0.16845 (19)	−0.00809 (18)	−0.00436 (14)	0.0301 (5)	
H22	0.0967	0.0316	−0.0362	0.036*	
C23	0.28514 (19)	−0.01504 (18)	−0.05205 (14)	0.0300 (5)	
H23	0.2931	0.0184	−0.1161	0.036*	
C24	0.38999 (19)	−0.07118 (17)	−0.00546 (13)	0.0262 (4)	
H24	0.4703	−0.0775	−0.0375	0.031*	
C25	0.37652 (17)	−0.11814 (16)	0.08832 (13)	0.0223 (4)	
C26	0.25873 (17)	−0.11432 (16)	0.13714 (13)	0.0227 (4)	
C27	0.25414 (17)	−0.17512 (16)	0.23418 (13)	0.0215 (4)	
C28	0.35753 (17)	−0.25970 (17)	0.27479 (13)	0.0223 (4)	
C29	0.5033 (2)	−0.39860 (18)	0.19582 (15)	0.0322 (5)	
H29A	0.4899	−0.4631	0.2476	0.039*	
H29B	0.5895	−0.4193	0.1689	0.039*	
H29C	0.4473	−0.3823	0.1500	0.039*	
C30	0.31849 (17)	−0.30032 (16)	0.36504 (13)	0.0225 (4)	
C31	0.38679 (19)	−0.39090 (18)	0.44001 (14)	0.0311 (5)	
H31A	0.4756	−0.4133	0.4194	0.037*	
H31B	0.3560	−0.4587	0.4575	0.037*	
H31C	0.3739	−0.3605	0.4926	0.037*	
C32	0.11075 (18)	−0.25551 (17)	0.45531 (13)	0.0252 (4)	
H32A	0.0379	−0.1858	0.4501	0.030*	
H32B	0.1516	−0.2658	0.5091	0.030*	
C33	0.06770 (17)	−0.36078 (17)	0.46880 (13)	0.0242 (4)	
C34	−0.10689 (17)	−0.36873 (17)	0.67866 (13)	0.0245 (4)	
H34	−0.1301	−0.4350	0.6822	0.029*	
C35	−0.14732 (17)	−0.31510 (17)	0.75402 (13)	0.0227 (4)	
C36	−0.21890 (17)	−0.35873 (18)	0.83283 (14)	0.0262 (4)	
C37	−0.25619 (18)	−0.3068 (2)	0.90357 (14)	0.0316 (5)	
H37	−0.3026	−0.3390	0.9573	0.038*	
C38	−0.22519 (19)	−0.2081 (2)	0.89518 (15)	0.0339 (5)	
H38	−0.2522	−0.1710	0.9426	0.041*	
C39	−0.15498 (19)	−0.16290 (19)	0.81798 (15)	0.0318 (5)	
H39	−0.1339	−0.0949	0.8125	0.038*	
C40	−0.11553 (18)	−0.21659 (18)	0.74900 (14)	0.0272 (4)	
H40	−0.0656	−0.1859	0.6969	0.033*	

### Atomic displacement parameters (Å^2^)

**Table:**

	*U*^11^	*U*^22^	*U*^33^	*U*^12^	*U*^13^	*U*^23^
Cl1	0.0357 (3)	0.0364 (3)	0.0268 (3)	−0.0187 (2)	0.0064 (2)	−0.0037 (2)
Cl2	0.0393 (3)	0.0396 (3)	0.0294 (3)	−0.0234 (2)	0.0074 (2)	−0.0053 (2)
S1	0.0320 (3)	0.0300 (3)	0.0205 (3)	−0.0019 (2)	0.0051 (2)	−0.0043 (2)
S2	0.0226 (2)	0.0284 (3)	0.0189 (3)	−0.0114 (2)	0.00193 (19)	−0.0018 (2)
O1	0.0310 (8)	0.0627 (11)	0.0270 (9)	−0.0042 (8)	0.0108 (7)	−0.0027 (8)
O2	0.0704 (11)	0.0260 (8)	0.0323 (9)	−0.0015 (8)	−0.0032 (8)	−0.0091 (7)
O3	0.0515 (9)	0.0381 (9)	0.0277 (8)	−0.0269 (7)	0.0168 (7)	−0.0175 (7)
O4	0.0253 (7)	0.0407 (9)	0.0231 (8)	−0.0080 (6)	0.0070 (6)	−0.0029 (7)
O5	0.0337 (8)	0.0335 (8)	0.0287 (8)	−0.0181 (6)	−0.0019 (6)	−0.0066 (7)
O6	0.0458 (9)	0.0357 (8)	0.0272 (8)	−0.0248 (7)	0.0162 (7)	−0.0172 (7)
N1	0.0224 (8)	0.0312 (9)	0.0190 (9)	−0.0097 (7)	0.0034 (7)	−0.0027 (7)
N2	0.0277 (9)	0.0294 (9)	0.0206 (9)	−0.0130 (7)	0.0013 (7)	−0.0048 (7)
N3	0.0270 (8)	0.0282 (9)	0.0162 (8)	−0.0137 (7)	0.0040 (7)	−0.0049 (7)
N4	0.0356 (9)	0.0314 (10)	0.0209 (9)	−0.0203 (8)	0.0084 (7)	−0.0114 (8)
N5	0.0273 (8)	0.0280 (9)	0.0207 (9)	−0.0103 (7)	0.0029 (7)	−0.0100 (7)
N6	0.0234 (8)	0.0243 (9)	0.0179 (8)	−0.0083 (7)	0.0015 (7)	−0.0014 (7)
N7	0.0257 (8)	0.0247 (9)	0.0198 (9)	−0.0113 (7)	0.0006 (7)	−0.0012 (7)
N8	0.0269 (8)	0.0238 (9)	0.0172 (8)	−0.0128 (7)	0.0039 (7)	−0.0039 (7)
N9	0.0338 (9)	0.0304 (9)	0.0218 (9)	−0.0191 (8)	0.0094 (7)	−0.0133 (8)
N10	0.0256 (8)	0.0270 (9)	0.0196 (9)	−0.0102 (7)	0.0037 (7)	−0.0094 (7)
C1	0.0289 (10)	0.0294 (11)	0.0258 (11)	−0.0132 (9)	−0.0026 (8)	−0.0064 (9)
C2	0.0347 (11)	0.0344 (12)	0.0267 (12)	−0.0134 (9)	−0.0100 (9)	−0.0016 (10)
C3	0.0451 (13)	0.0373 (13)	0.0197 (11)	−0.0202 (10)	−0.0026 (9)	−0.0003 (9)
C4	0.0370 (12)	0.0323 (12)	0.0217 (11)	−0.0115 (9)	0.0030 (9)	−0.0034 (9)
C5	0.0313 (11)	0.0246 (11)	0.0202 (11)	−0.0106 (8)	0.0017 (8)	−0.0038 (9)
C6	0.0289 (10)	0.0221 (10)	0.0218 (10)	−0.0132 (8)	−0.0003 (8)	−0.0050 (8)
C7	0.0246 (10)	0.0222 (10)	0.0187 (10)	−0.0110 (8)	0.0019 (8)	−0.0055 (8)
C8	0.0229 (10)	0.0250 (10)	0.0197 (10)	−0.0096 (8)	0.0026 (8)	−0.0060 (8)
C9	0.0299 (11)	0.0446 (14)	0.0354 (13)	−0.0199 (10)	0.0006 (10)	−0.0073 (11)
C10	0.0267 (10)	0.0251 (10)	0.0205 (10)	−0.0115 (8)	0.0010 (8)	−0.0060 (8)
C11	0.0361 (12)	0.0405 (13)	0.0215 (11)	−0.0138 (10)	−0.0023 (9)	−0.0001 (10)
C12	0.0308 (10)	0.0299 (11)	0.0210 (11)	−0.0167 (9)	0.0093 (8)	−0.0093 (9)
C13	0.0277 (10)	0.0305 (11)	0.0202 (10)	−0.0140 (9)	0.0048 (8)	−0.0074 (9)
C14	0.0263 (10)	0.0313 (11)	0.0216 (11)	−0.0148 (9)	0.0048 (8)	−0.0079 (9)
C15	0.0240 (10)	0.0287 (11)	0.0180 (10)	−0.0081 (8)	−0.0003 (8)	−0.0055 (8)
C16	0.0241 (10)	0.0322 (11)	0.0208 (10)	−0.0097 (8)	0.0006 (8)	−0.0052 (9)
C17	0.0326 (11)	0.0463 (14)	0.0213 (11)	−0.0135 (10)	0.0074 (9)	−0.0109 (10)
C18	0.0384 (12)	0.0500 (15)	0.0286 (12)	−0.0148 (11)	0.0080 (10)	−0.0218 (11)
C19	0.0415 (12)	0.0390 (13)	0.0314 (12)	−0.0148 (10)	0.0017 (10)	−0.0169 (10)
C20	0.0334 (11)	0.0360 (12)	0.0226 (11)	−0.0170 (9)	0.0061 (9)	−0.0088 (9)
C21	0.0264 (10)	0.0281 (11)	0.0258 (11)	−0.0138 (8)	−0.0021 (8)	−0.0033 (9)
C22	0.0327 (11)	0.0336 (12)	0.0246 (11)	−0.0143 (9)	−0.0088 (9)	−0.0005 (9)
C23	0.0386 (12)	0.0335 (12)	0.0187 (10)	−0.0171 (9)	−0.0024 (9)	−0.0018 (9)
C24	0.0315 (10)	0.0271 (11)	0.0200 (10)	−0.0136 (9)	0.0012 (8)	−0.0033 (9)
C25	0.0266 (10)	0.0212 (10)	0.0189 (10)	−0.0103 (8)	−0.0004 (8)	−0.0027 (8)
C26	0.0281 (10)	0.0219 (10)	0.0192 (10)	−0.0125 (8)	0.0012 (8)	−0.0037 (8)
C27	0.0232 (9)	0.0227 (10)	0.0189 (10)	−0.0116 (8)	0.0013 (8)	−0.0030 (8)
C28	0.0224 (9)	0.0261 (10)	0.0178 (10)	−0.0111 (8)	0.0015 (8)	−0.0031 (8)
C29	0.0386 (12)	0.0261 (11)	0.0296 (12)	−0.0097 (9)	0.0006 (9)	−0.0066 (9)
C30	0.0250 (10)	0.0239 (10)	0.0199 (10)	−0.0115 (8)	0.0016 (8)	−0.0057 (8)
C31	0.0331 (11)	0.0323 (12)	0.0201 (11)	−0.0096 (9)	0.0011 (9)	0.0008 (9)
C32	0.0301 (10)	0.0269 (11)	0.0197 (10)	−0.0145 (8)	0.0084 (8)	−0.0080 (9)
C33	0.0255 (10)	0.0277 (11)	0.0201 (10)	−0.0122 (8)	0.0044 (8)	−0.0066 (9)
C34	0.0267 (10)	0.0280 (11)	0.0217 (10)	−0.0141 (8)	0.0042 (8)	−0.0083 (9)
C35	0.0208 (9)	0.0285 (11)	0.0174 (10)	−0.0079 (8)	0.0012 (8)	−0.0055 (8)
C36	0.0222 (10)	0.0335 (12)	0.0219 (11)	−0.0097 (8)	0.0003 (8)	−0.0062 (9)
C37	0.0269 (10)	0.0478 (14)	0.0185 (11)	−0.0136 (10)	0.0051 (8)	−0.0086 (10)
C38	0.0294 (11)	0.0512 (14)	0.0263 (12)	−0.0114 (10)	0.0033 (9)	−0.0215 (11)
C39	0.0320 (11)	0.0368 (12)	0.0314 (12)	−0.0124 (9)	0.0006 (9)	−0.0155 (10)
C40	0.0273 (10)	0.0329 (11)	0.0234 (11)	−0.0131 (9)	0.0030 (8)	−0.0094 (9)

### Geometric parameters (Å, °)

**Table:**

Cl1—C16	1.744 (2)	C11—H11C	0.9800
Cl2—C36	1.744 (2)	C12—C13	1.521 (3)
S1—O2	1.4276 (17)	C12—H12A	0.9900
S1—O1	1.4298 (17)	C12—H12B	0.9900
S1—N1	1.6481 (17)	C14—C15	1.460 (3)
S1—C5	1.769 (2)	C14—H14	0.9500
S2—O4	1.4295 (14)	C15—C20	1.395 (3)
S2—O5	1.4315 (15)	C15—C16	1.398 (3)
S2—N6	1.6440 (16)	C16—C17	1.388 (3)
S2—C25	1.7734 (19)	C17—C18	1.384 (3)
O3—C13	1.225 (2)	C17—H17	0.9500
O6—C33	1.228 (2)	C18—C19	1.384 (3)
N1—C8	1.430 (2)	C18—H18	0.9500
N1—C9	1.483 (3)	C19—C20	1.378 (3)
N2—C7	1.334 (2)	C19—H19	0.9500
N2—N3	1.363 (2)	C20—H20	0.9500
N3—C10	1.365 (2)	C21—C22	1.386 (3)
N3—C12	1.448 (2)	C21—C26	1.393 (3)
N4—C13	1.343 (2)	C21—H21	0.9500
N4—N5	1.380 (2)	C22—C23	1.389 (3)
N4—H04	0.8800	C22—H22	0.9500
N5—C14	1.278 (2)	C23—C24	1.387 (3)
N6—C28	1.430 (2)	C23—H23	0.9500
N6—C29	1.484 (3)	C24—C25	1.388 (3)
N7—C27	1.338 (2)	C24—H24	0.9500
N7—N8	1.362 (2)	C25—C26	1.410 (3)
N8—C30	1.361 (2)	C26—C27	1.460 (3)
N8—C32	1.446 (2)	C27—C28	1.405 (3)
N9—C33	1.341 (2)	C28—C30	1.373 (3)
N9—N10	1.382 (2)	C29—H29A	0.9800
N9—H09	0.8800	C29—H29B	0.9800
N10—C34	1.274 (2)	C29—H29C	0.9800
C1—C2	1.383 (3)	C30—C31	1.483 (3)
C1—C6	1.394 (3)	C31—H31A	0.9800
C1—H1	0.9500	C31—H31B	0.9800
C2—C3	1.388 (3)	C31—H31C	0.9800
C2—H2	0.9500	C32—C33	1.521 (3)
C3—C4	1.383 (3)	C32—H32A	0.9900
C3—H3	0.9500	C32—H32B	0.9900
C4—C5	1.390 (3)	C34—C35	1.466 (3)
C4—H4	0.9500	C34—H34	0.9500
C5—C6	1.408 (3)	C35—C40	1.396 (3)
C6—C7	1.461 (3)	C35—C36	1.401 (3)
C7—C8	1.407 (3)	C36—C37	1.388 (3)
C8—C10	1.371 (3)	C37—C38	1.381 (3)
C9—H9A	0.9800	C37—H37	0.9500
C9—H9B	0.9800	C38—C39	1.383 (3)
C9—H9C	0.9800	C38—H38	0.9500
C10—C11	1.488 (3)	C39—C40	1.379 (3)
C11—H11A	0.9800	C39—H39	0.9500
C11—H11B	0.9800	C40—H40	0.9500
			
O2—S1—O1	119.67 (11)	C20—C15—C14	120.97 (18)
O2—S1—N1	107.75 (9)	C16—C15—C14	121.48 (18)
O1—S1—N1	107.62 (10)	C17—C16—C15	121.44 (19)
O2—S1—C5	106.56 (10)	C17—C16—Cl1	118.40 (16)
O1—S1—C5	109.55 (10)	C15—C16—Cl1	120.16 (15)
N1—S1—C5	104.71 (9)	C18—C17—C16	119.5 (2)
O4—S2—O5	119.73 (9)	C18—C17—H17	120.3
O4—S2—N6	108.16 (9)	C16—C17—H17	120.3
O5—S2—N6	107.65 (8)	C17—C18—C19	120.1 (2)
O4—S2—C25	109.04 (9)	C17—C18—H18	119.9
O5—S2—C25	107.54 (9)	C19—C18—H18	119.9
N6—S2—C25	103.53 (8)	C20—C19—C18	120.0 (2)
C8—N1—C9	113.18 (16)	C20—C19—H19	120.0
C8—N1—S1	110.51 (12)	C18—C19—H19	120.0
C9—N1—S1	115.83 (13)	C19—C20—C15	121.4 (2)
C7—N2—N3	103.85 (15)	C19—C20—H20	119.3
N2—N3—C10	113.56 (15)	C15—C20—H20	119.3
N2—N3—C12	119.49 (15)	C22—C21—C26	120.25 (18)
C10—N3—C12	126.94 (17)	C22—C21—H21	119.9
C13—N4—N5	121.66 (16)	C26—C21—H21	119.9
C13—N4—H04	119.2	C21—C22—C23	121.22 (19)
N5—N4—H04	119.2	C21—C22—H22	119.4
C14—N5—N4	113.66 (16)	C23—C22—H22	119.4
C28—N6—C29	115.32 (15)	C24—C23—C22	119.52 (19)
C28—N6—S2	109.81 (12)	C24—C23—H23	120.2
C29—N6—S2	115.80 (13)	C22—C23—H23	120.2
C27—N7—N8	103.62 (15)	C23—C24—C25	119.43 (19)
C30—N8—N7	113.79 (15)	C23—C24—H24	120.3
C30—N8—C32	126.45 (17)	C25—C24—H24	120.3
N7—N8—C32	119.58 (15)	C24—C25—C26	121.56 (17)
C33—N9—N10	121.36 (16)	C24—C25—S2	120.38 (15)
C33—N9—H09	119.3	C26—C25—S2	118.03 (14)
N10—N9—H09	119.3	C21—C26—C25	117.97 (17)
C34—N10—N9	113.79 (16)	C21—C26—C27	124.23 (17)
C2—C1—C6	120.39 (19)	C25—C26—C27	117.72 (17)
C2—C1—H1	119.8	N7—C27—C28	111.17 (16)
C6—C1—H1	119.8	N7—C27—C26	126.21 (17)
C1—C2—C3	121.03 (19)	C28—C27—C26	122.61 (17)
C1—C2—H2	119.5	C30—C28—C27	106.59 (16)
C3—C2—H2	119.5	C30—C28—N6	128.76 (17)
C4—C3—C2	119.8 (2)	C27—C28—N6	124.65 (17)
C4—C3—H3	120.1	N6—C29—H29A	109.5
C2—C3—H3	120.1	N6—C29—H29B	109.5
C3—C4—C5	119.4 (2)	H29A—C29—H29B	109.5
C3—C4—H4	120.3	N6—C29—H29C	109.5
C5—C4—H4	120.3	H29A—C29—H29C	109.5
C4—C5—C6	121.53 (19)	H29B—C29—H29C	109.5
C4—C5—S1	119.17 (15)	N8—C30—C28	104.83 (16)
C6—C5—S1	119.13 (15)	N8—C30—C31	124.68 (17)
C1—C6—C5	117.93 (18)	C28—C30—C31	130.49 (17)
C1—C6—C7	123.84 (18)	C30—C31—H31A	109.5
C5—C6—C7	118.22 (17)	C30—C31—H31B	109.5
N2—C7—C8	111.11 (16)	H31A—C31—H31B	109.5
N2—C7—C6	125.81 (17)	C30—C31—H31C	109.5
C8—C7—C6	123.06 (16)	H31A—C31—H31C	109.5
C10—C8—C7	106.71 (16)	H31B—C31—H31C	109.5
C10—C8—N1	128.69 (17)	N8—C32—C33	111.10 (15)
C7—C8—N1	124.34 (17)	N8—C32—H32A	109.4
N1—C9—H9A	109.5	C33—C32—H32A	109.4
N1—C9—H9B	109.5	N8—C32—H32B	109.4
H9A—C9—H9B	109.5	C33—C32—H32B	109.4
N1—C9—H9C	109.5	H32A—C32—H32B	108.0
H9A—C9—H9C	109.5	O6—C33—N9	121.04 (18)
H9B—C9—H9C	109.5	O6—C33—C32	122.09 (17)
N3—C10—C8	104.75 (17)	N9—C33—C32	116.87 (16)
N3—C10—C11	124.02 (17)	N10—C34—C35	120.56 (18)
C8—C10—C11	131.22 (18)	N10—C34—H34	119.7
C10—C11—H11A	109.5	C35—C34—H34	119.7
C10—C11—H11B	109.5	C40—C35—C36	117.36 (18)
H11A—C11—H11B	109.5	C40—C35—C34	120.32 (18)
C10—C11—H11C	109.5	C36—C35—C34	122.32 (18)
H11A—C11—H11C	109.5	C37—C36—C35	121.51 (19)
H11B—C11—H11C	109.5	C37—C36—Cl2	118.54 (16)
N3—C12—C13	112.17 (16)	C35—C36—Cl2	119.96 (15)
N3—C12—H12A	109.2	C38—C37—C36	119.40 (19)
C13—C12—H12A	109.2	C38—C37—H37	120.3
N3—C12—H12B	109.2	C36—C37—H37	120.3
C13—C12—H12B	109.2	C37—C38—C39	120.28 (19)
H12A—C12—H12B	107.9	C37—C38—H38	119.9
O3—C13—N4	121.57 (18)	C39—C38—H38	119.9
O3—C13—C12	122.80 (17)	C40—C39—C38	120.0 (2)
N4—C13—C12	115.63 (17)	C40—C39—H39	120.0
N5—C14—C15	120.76 (18)	C38—C39—H39	120.0
N5—C14—H14	119.6	C39—C40—C35	121.42 (19)
C15—C14—H14	119.6	C39—C40—H40	119.3
C20—C15—C16	117.54 (18)	C35—C40—H40	119.3
			
O2—S1—N1—C8	64.64 (15)	C20—C15—C16—Cl1	179.16 (15)
O1—S1—N1—C8	−165.04 (13)	C14—C15—C16—Cl1	−0.5 (3)
C5—S1—N1—C8	−48.53 (15)	C15—C16—C17—C18	−0.6 (3)
O2—S1—N1—C9	−164.98 (14)	Cl1—C16—C17—C18	179.75 (17)
O1—S1—N1—C9	−34.66 (17)	C16—C17—C18—C19	0.9 (3)
C5—S1—N1—C9	81.85 (15)	C17—C18—C19—C20	−0.1 (3)
C7—N2—N3—C10	1.0 (2)	C18—C19—C20—C15	−1.1 (3)
C7—N2—N3—C12	179.57 (16)	C16—C15—C20—C19	1.3 (3)
C13—N4—N5—C14	176.32 (19)	C14—C15—C20—C19	−179.00 (19)
O4—S2—N6—C28	−167.43 (12)	C26—C21—C22—C23	1.4 (3)
O5—S2—N6—C28	61.86 (14)	C21—C22—C23—C24	−1.1 (3)
C25—S2—N6—C28	−51.83 (14)	C22—C23—C24—C25	−0.9 (3)
O4—S2—N6—C29	−34.67 (16)	C23—C24—C25—C26	2.6 (3)
O5—S2—N6—C29	−165.37 (13)	C23—C24—C25—S2	−175.41 (15)
C25—S2—N6—C29	80.94 (15)	O4—S2—C25—C24	−26.45 (19)
C27—N7—N8—C30	0.4 (2)	O5—S2—C25—C24	104.80 (16)
C27—N7—N8—C32	−175.10 (15)	N6—S2—C25—C24	−141.43 (16)
C33—N9—N10—C34	−175.35 (18)	O4—S2—C25—C26	155.45 (15)
C6—C1—C2—C3	0.0 (3)	O5—S2—C25—C26	−73.30 (16)
C1—C2—C3—C4	0.8 (3)	N6—S2—C25—C26	40.48 (17)
C2—C3—C4—C5	−1.0 (3)	C22—C21—C26—C25	0.3 (3)
C3—C4—C5—C6	0.4 (3)	C22—C21—C26—C27	−176.28 (18)
C3—C4—C5—S1	−174.74 (16)	C24—C25—C26—C21	−2.3 (3)
O2—S1—C5—C4	95.97 (18)	S2—C25—C26—C21	175.79 (14)
O1—S1—C5—C4	−34.8 (2)	C24—C25—C26—C27	174.49 (17)
N1—S1—C5—C4	−150.01 (16)	S2—C25—C26—C27	−7.4 (2)
O2—S1—C5—C6	−79.25 (17)	N8—N7—C27—C28	−0.7 (2)
O1—S1—C5—C6	149.94 (16)	N8—N7—C27—C26	178.13 (17)
N1—S1—C5—C6	34.76 (18)	C21—C26—C27—N7	−19.1 (3)
C2—C1—C6—C5	−0.6 (3)	C25—C26—C27—N7	164.35 (18)
C2—C1—C6—C7	−179.12 (18)	C21—C26—C27—C28	159.56 (19)
C4—C5—C6—C1	0.4 (3)	C25—C26—C27—C28	−17.0 (3)
S1—C5—C6—C1	175.55 (14)	N7—C27—C28—C30	0.7 (2)
C4—C5—C6—C7	179.03 (18)	C26—C27—C28—C30	−178.12 (17)
S1—C5—C6—C7	−5.9 (2)	N7—C27—C28—N6	−179.99 (16)
N3—N2—C7—C8	−0.2 (2)	C26—C27—C28—N6	1.2 (3)
N3—N2—C7—C6	−178.90 (17)	C29—N6—C28—C30	83.4 (2)
C1—C6—C7—N2	−14.4 (3)	S2—N6—C28—C30	−143.56 (18)
C5—C6—C7—N2	167.13 (18)	C29—N6—C28—C27	−95.7 (2)
C1—C6—C7—C8	167.05 (18)	S2—N6—C28—C27	37.3 (2)
C5—C6—C7—C8	−11.5 (3)	N7—N8—C30—C28	0.0 (2)
N2—C7—C8—C10	−0.7 (2)	C32—N8—C30—C28	175.16 (17)
C6—C7—C8—C10	178.07 (17)	N7—N8—C30—C31	−179.45 (17)
N2—C7—C8—N1	173.85 (17)	C32—N8—C30—C31	−4.3 (3)
C6—C7—C8—N1	−7.4 (3)	C27—C28—C30—N8	−0.4 (2)
C9—N1—C8—C10	82.0 (2)	N6—C28—C30—N8	−179.69 (18)
S1—N1—C8—C10	−146.19 (18)	C27—C28—C30—C31	179.02 (19)
C9—N1—C8—C7	−91.3 (2)	N6—C28—C30—C31	−0.2 (3)
S1—N1—C8—C7	40.5 (2)	C30—N8—C32—C33	−76.9 (2)
N2—N3—C10—C8	−1.5 (2)	N7—N8—C32—C33	97.98 (19)
C12—N3—C10—C8	−179.88 (17)	N10—N9—C33—O6	176.29 (17)
N2—N3—C10—C11	177.98 (18)	N10—N9—C33—C32	−3.5 (3)
C12—N3—C10—C11	−0.4 (3)	N8—C32—C33—O6	−2.4 (3)
C7—C8—C10—N3	1.2 (2)	N8—C32—C33—N9	177.41 (17)
N1—C8—C10—N3	−172.99 (18)	N9—N10—C34—C35	179.12 (16)
C7—C8—C10—C11	−178.1 (2)	N10—C34—C35—C40	1.0 (3)
N1—C8—C10—C11	7.6 (4)	N10—C34—C35—C36	−179.33 (19)
N2—N3—C12—C13	−87.3 (2)	C40—C35—C36—C37	−0.5 (3)
C10—N3—C12—C13	91.0 (2)	C34—C35—C36—C37	179.91 (18)
N5—N4—C13—O3	−175.86 (18)	C40—C35—C36—Cl2	179.38 (14)
N5—N4—C13—C12	4.7 (3)	C34—C35—C36—Cl2	−0.3 (3)
N3—C12—C13—O3	3.9 (3)	C35—C36—C37—C38	1.9 (3)
N3—C12—C13—N4	−176.66 (17)	Cl2—C36—C37—C38	−177.96 (16)
N4—N5—C14—C15	−178.77 (16)	C36—C37—C38—C39	−1.6 (3)
N5—C14—C15—C20	0.9 (3)	C37—C38—C39—C40	−0.1 (3)
N5—C14—C15—C16	−179.43 (19)	C38—C39—C40—C35	1.5 (3)
C20—C15—C16—C17	−0.5 (3)	C36—C35—C40—C39	−1.3 (3)
C14—C15—C16—C17	179.85 (18)	C34—C35—C40—C39	178.39 (19)

### Hydrogen-bond geometry (Å, °)

**Table:**

*D*—H···*A*	*D*—H	H···*A*	*D*···*A*	*D*—H···*A*
C14—H14···Cl1	0.95	2.64	3.048 (2)	106.
C34—H34···Cl2	0.95	2.67	3.068 (2)	106.
N4—H04···O3^i^	0.88	2.01	2.883 (2)	172.
N9—H09···O6^ii^	0.88	1.96	2.837 (2)	173.
C18—H18···O1^iii^	0.95	2.55	3.204 (3)	127.
C29—H29A···O2^iv^	0.98	2.31	3.266 (3)	165.
C38—H38···O4^v^	0.95	2.54	3.233 (2)	130.
C9—H9B···O1	0.98	2.49	2.843 (3)	101.
C29—H29B···O4	0.98	2.51	2.851 (3)	100.

Symmetry codes: (i) −*x*, −*y*+1, −*z*; (ii) −*x*, −*y*−1, −*z*+1; (iii) *x*+1, *y*, *z*−1; (iv) *x*+1, *y*−1, *z*; (v) *x*−1, *y*, *z*+1.
